# Clinicopathological analysis of polyploid diffuse large B-cell lymphoma

**DOI:** 10.1371/journal.pone.0194525

**Published:** 2018-04-11

**Authors:** Joji Shimono, Hiroaki Miyoshi, Junichi Kiyasu, Tomohiko Kamimura, Tetsuya Eto, Takuto Miyagishima, Koji Nagafuji, Masao Seto, Takanori Teshima, Koichi Ohshima

**Affiliations:** 1 Department of Pathology, Kurume University, School of Medicine, Kurume, Japan; 2 Department of Hematology, Hokkaido University Faculty of Medicine, Sapporo, Japan; 3 Department of Hematology, Iizuka hospital, Iizuka, Japan; 4 Department of Hematology, Harasanshin Hospital, Fukuoka, Japan; 5 Department of Hematology, Hamanomachi Hospital, Fukuoka, Japan; 6 Department of Hematology, Kushiro Rosai Hospital, Kushiro, Japan; 7 Department of Hematology, Kurume University, School of Medicine, Kurume, Japan; Hokkaido Daigaku, JAPAN

## Abstract

Polyploid chromosomes are those with more than two sets of homologous chromosomes. Polyploid chromosomal abnormalities are observed in various malignant tumors. The prognosis in such cases is generally poor. However, there are no studies examining the prognosis of diffuse large B-cell lymphoma (DLBCL) with polyploid chromosomal abnormalities. Therefore, we statistically compared the clinicopathological features between polyploid DLBCL and DLBCL without polyploid abnormalities. Herein, 51 polyploid DLBCL and 53 control (without polyploid chromosomal abnormalities) cases were examined. G-banding method was employed to define polyploidy by cytogenetic analysis. Subsequently, flow cytometric immunophenotyping and immunohistochemical staining were performed. Polyploid DLBCL was defined as DLBCL with either near-tetraploid or greater number of chromosomes, as detected by the G-band. In a survival analysis, a significantly worse overall survival (OS) was observed for polyploid DLBCL (*p* = 0.04; *p* = 0.02 in cases who received R-CHOP regimens). In a multivariate analysis of OS, polyploid chromosomal abnormalities were an independent prognostic factor. Our results suggest that polyploid chromosomal abnormalities detected through G-band may represent a new poor prognostic factor for DLBCL.

## Introduction

Diffuse large B-cell lymphoma (DLBCL) is the most frequent type of B-cell lymphoma, accounting for 30%-40% of non-Hodgkin’s lymphoma [1]. The biological properties, genetic mutations, immune phenotypes, and cell morphology of DLBCL are varied, and therefore, DLBCL is considered a heterogeneous group [2]. Chromosomal translocation of immunoglobulin genes, such as *c-MYC* and *BCL2*, are widely observed as chromosomal abnormalities in DLBCL. In addition, these chromosomal translocations are associated with poor prognosis in DLBCL [3,4].

Polyploidy refers to the existence of more than two sets of homologous chromosomes within a cell. Abnormalities in the cell cycle, re-replication of DNA, and cell division are some factors that cause polyploidy [5,6]. In normal tissue, chromosome polyploidization can be observed in placental trophoblasts and megakaryocytes during the differentiation process. Polyploid chromosomal abnormalities are also seen in 6.9% of malignant tumors, and are associated with poor prognosis in acute myeloid leukemia, breast cancer, ovarian cancer, and colon cancer [7–10].

We had previously examined the histological features of 16 cases of DLBCL in which polyploid chromosomal abnormalities were observed by using the G-band method [11]. DLBCL with polyploid chromosomal abnormalities was observed in 2.9% of the cases. Unique histological characteristics, Hodgkin’s-like giant cells and multilobated cells, were observed in polyploid DLBCL [11]. However, to the best of our knowledge, there are no studies comparing the clinicopathological features between polyploid DLBCL and DLBCL without polyploid chromosomal abnormalities.

Therefore, in the present study, we statistically compared the clinicopathological features between polyploid DLBCL and DLBCL without polyploid abnormalities.

## Materials and methods

### Patients and tissue samples

We reviewed 51 cases of polyploid DLBCL, not otherwise specified (NOS) from 2008 to 2014 in the Department of Pathology, Kurume University. The karyotypes of the polyploid DLBCL cases are shown in S1 Table. Additionally, 53 cases of DLBCL, NOS were extracted as control cases from our previous study [12]. The control cases did not have polyploid abnormalities, validated by using the G-banding method (S2 Table). Paraffin-embedded tissues were used for diagnosis and immunohistochemical staining (IHS). All cases were reviewed by experienced hematopathologists (OK and MH) and were diagnosed according to the World Health Organization (WHO) classification [13]. The use of materials and clinical information were approved by the Research Ethics Committee of Kurume University and were in accordance with the Declaration of Helsinki. All data were fully anonymized.

### Definition of polyploidy in cytogenetic analysis

The G-banding method was performed for cytogenetic analysis. Karyotypes were reviewed according to the International System for Human Cytogenetics Nomenclature (ISCN 2013). We defined DLBCL cases with near-tetraploid or greater number of chromosomes as polyploid DLBCL as per our previous study [11]. According to ISCN 2013, chromosomal numbers of 81–103 was defined as near-tetraploid and chromosomal numbers of 104–126 was defined as near-pentaploid, while DLBCL with a chromosomal number of >127 was not observed in this study.

### Flow cytometric immunophenotyping analysis

For flow cytometric immunophenotyping analysis, unfixed tissues were pulverized to prepare a cell suspension. The samples were centrifuged at 1800 rpm for 5 min, and washed with 5 mL of 10% phosphate-buffered saline (PBS) after discarding the suspension twice. The cells were then resuspended with CD45 (J.33; Becton-Dickinson, Mountain View, CA, USA) and then with the other antibodies as follows at 4°C for 30 min. The hemolytic agents were then added, and the samples were centrifuged again at 1800 rpm for 5 min, and then washed with 5 mL of 10% PBS after discarding the suspension twice. Flow cytometric immunophenotyping analysis was then performed with a flow cytometer (FACS-Calibur, Becton-Dickinson) and the data were analyzed with the Cell Quest software program (Becton-Dickinson). The cells were gated according to forward scatter (FSC) and side scatter (SSC), and tumor cells were further gated according to CD45 and SSC. The gated area with tumor cells was then analyzed by fluorescein isothiocyanate (FITC) and phycoerythrin (PE) detection conjugated to the antibodies for the relevant markers. Information of the antibodies is summarized in S3 Table. Evaluation of live cells was conducted using propidium iodide (PI) staining (500 μg/mL). Mouse IgG1-FITC (Becton-Dickinson) and Mouse IgG1-PE (Becton-Dickinson) were used as negative controls.

### Immunohistochemical staining

Immunohistochemical staining (IHC) was carried out using 2.5-μm-thick, formalin-fixed, paraffin-embedded tissue sections for all cases. The slides were deparaffinized with xylene followed by ethanol. After rehydration with water, antigen retrieval was performed with antibody-specific buffer in a microwave oven. Endogenous peroxidase activity was blocked by incubating in 3% hydrogen peroxide for 5 min. The slides were then incubated with each antibody and EnVision1 System horseradish peroxide-labeled anti-mouse polymer (Dakocytomation) for 30 min. Visualization was performed using diaminobenzidine for 5 min. The slides were counterstained with hematoxylin, dehydrated with ethanol, and mounted under coverslips. Information of the antibodies is provided in S3 Table. If >30% of the neoplastic cells were immunostained (except for p53 staining), the case was defined as positive. According to the results of a previous study, only cases showing a strong immunostaining intensity, in which the anti-p53 antibody immunostained > 50% of the neoplastic cells, were defined as p53-positive [14]. When CD5-positive DLBCL was detected, cyclin D1 (SP4) (ThermoScientific, Runcorn, UK) staining was performed to exclude mantle cell lymphoma, an aggressive variant.

### Quantification of various markers in CD20-positive tumor cells

The positive ratios of several markers (CD5, CD10, CD30, BCL2, BCL6, and MUM1) were examined for the CD20-positive tumor cells using flow cytometry (CD5, CD10, and CD30) and IHC (BCL2, BCL6, and MUM1). The proportion of cells positive for each marker was divided by the CD20-positive cell ratio in the gated area of tumor cells (for flow cytometry) or positive tumor cells (for IHC).

### *In situ* hybridization for Epstein-Barr virus (EBV)-encoded RNA

EBV was detected by *in situ* hybridization with a fluorescein-conjugated EBV peptide nuclei acid (PNA) probe kit (Dakocytomation) according to the manufacturer’s instructions. This probe was complementary to the two nuclear EBER RNAs encoded by EBV. If >30% of the neoplastic cells were immunostained, the case was defined as positive.

### Fluorescence in situ hybridization (FISH) analysis

The following probes were used for FISH analysis according to previously published protocols [15, 16]: *MYC* FISH DNA Probe; Split Signal (Abbott Molecular, Abbott Park, IL, USA); the Vysis LSI *IGH*/*BCL2* Dual Color, Dual Fusion Translocation Probe (Abbott Molecular); and the Vysis LSI *BCL6* Dual Color Break Apart Rearrangement Probe (Abbott Molecular). Shortly after deparaffinization and dehydration, the specimens were incubated in 2X saline-sodium citrate (SSC) buffer at 80°C for 30 min and digested with protease (25–28°C, 10 min). After post-fixation and dehydration, the probe was applied to the slide under a coverslip and left to hybridize (95°C, 5 min; 37°C, 16 h). Hybridized slides were washed and air-dried before counterstaining with 4′,6-diamidino-2 phenylindole (DAPI) for fluorescence microscopy analysis. The cut-off values for positivity were the presence of signals in 1% of cells in a cell suspension and in 5% of cells, based on laboratory-established thresholds.

### Statistical analysis

Clinicopathological characteristics of the patients were compared by using the chi-squared test or Fisher’s two-sided exact test, as needed. The end-point of overall survival (OS) was defined as the time of death due to DLBCL. The end-point of progression free survival (PFS) was defined as the time of relapse due to DLBCL. Survival curves of OS and PFS were calculated using the Kaplan–Meier method. A log-rank test was used to compare survival curves. Univariate and multivariate Cox proportional regression models were used to evaluate the proposed prognostic factors. *P* < 0.05 was considered statistically significant. JMP version 11.0 was used in all analyses.

## Results

### Clinicopathological properties

**Fig 1:**
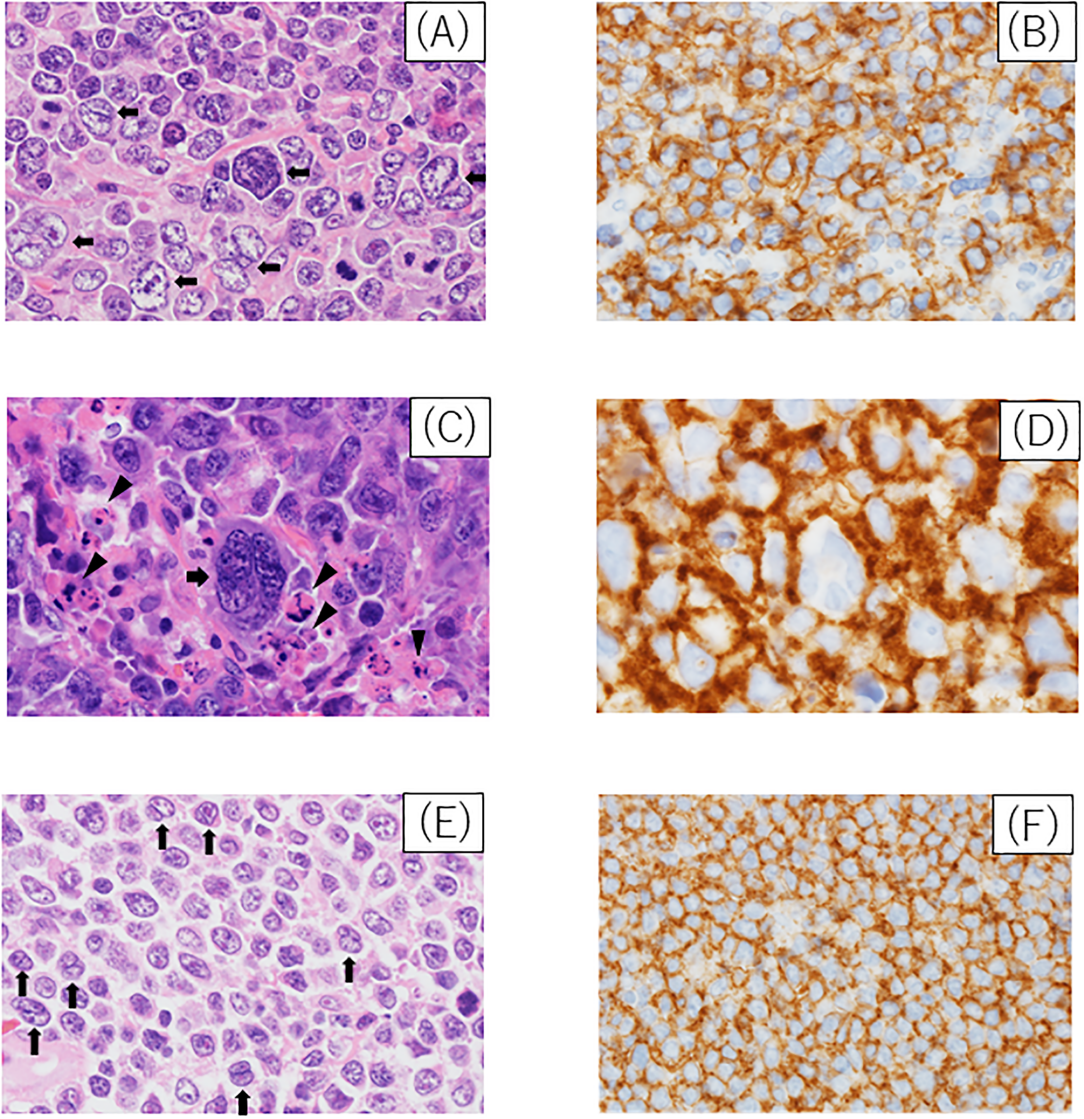
Histology of polyploid diffuse large B-cell lymphoma (DLBCL). (A) HE staining, large tumor cells (black arrows). (B) Tumor cells were positive for CD20. (C) HE staining, multilobated tumor cells (black arrows) with many apoptotic cells (arrow heads). (D) Tumor cells were positive for CD20. (E) HE staining, multilobated medium-sized cells (black arrows). (F) Tumor cells were positive for CD20.

We investigated a comparison with control DLBCL to clarify the clinicopathological properties of polyploid DLBCL. Table 1 shows the clinical features of the polyploid DLBCL and control DLBCL cases. No significant differences were observed between the two groups with respect to age, sex, performance status (PS), number of extranodal infiltration sites > 1, B symptoms, lactate dehydrogenase elevation, or advanced stage. Further, no significant differences were observed between the groups with respect to known poor prognostic factors, including the International Prognostic Index (IPI). Table 2 shows the clinical outcomes and therapy of the two case groups. No significant difference was noted between the two groups with respect to the treatment strategy and response to initial therapy. R-CHOP (rituximab, cyclophosphamide, doxorubicin, vincristine, and prednisone) or an R-CHOP-like regimen was carried out in 88.2% of the polyploid DLBCL cases (45/51) and in 79.2% (42/53) of the control DLBCL cases, with no significant difference (*p* = 0.21). Fig 1 shows the pathological features of the polyploid DLBCL cases. Consistent with our previous study [11], histological findings of Hodgkin’s-like giant cells and multilobated cells were observed in cases of polyploid DLBCL.

**Table 1:** Clinical features of polyploid DLBCL and control DLBCL.

Clinical features	Polyploid cases (N = 51)		Control cases (N = 53)		*p*-value
	No.	%	No.	%	
Age. Years					
Median	69		72		
Range	36–84		22–93		
Sex					
Male	29/51	56.9	23/53	43.4	0.17
Female	22/51	43.1	30/53	55.6	
ECOG PS > 1	12/51	23.5	15/53	28.3	0.58
Extranodal infiltration site > 1	6/51	11.8	9/53	17.0	0.45
B symptoms	11/51	21.6	10/53	18.9	0.73
Elevated LDH	36/51	70.6	39/53	73.6	0.73
Ann Arbor Stage > 2	36/51	70.6	36/53	67.9	0.77
IPI score					
Low/Low int	24/51	47.1	19/53	35.8	0.25
High int/High	27/51	52.9	34/53	54.2	
Median follow up period (Months)	27 months (1–86 months)		37 months (0.2–79.5 months)		

PS, performance status; LDH, lactate dehydrogenase; IPI, international prognostic index;

Low int, low intermediate risk; High int, high intermediate risk;

**Table 2:** Clinical outocomes and therapy of polyploid DLBCL and control DLBCL.

Clinical features	Polyploid cases (N = 51)		Control cases (N = 53)		*p*-value
	No.	%	No.	%	
Type of initial therapy					
Chemotherapy	48/51	94.1	46/53	86.8	0.32*
R-CHOP/R-CHOP like regimen	45/48	93.8	43/46	93.5	1.00*
Other	3/48	6.2	3/46	6.5	1.00*
Radiation therapy	10/51	19.6	9/53	17.0	0.73
Radiation therapy only	0/10	0.0	3/9	33.3	0.24*
No therapy	3/51	5.9	4/53	7.5	1.00*
Response to initial therapy					
CR	31/48	64.6	37/49	75.5	0.24
PR	10/48	20.8	6/49	12.2	0.25
SD	1/48	2.1	2/49	4.1	1.00*
PD	6/48	12.5	4/49	8.2.	0.52*
Not evaluable	3/51	5.9	4/53	7.5	1.00*

R-CHOP, rituximab, cyclophosphamide, doxorubicin, vincristine, and prednisone;

PBSCT, peripheral blood stem cell transplantation; CR, complete response; PR, partial response; SD, stable disease; PD, progressive disease.

*Fisher’s exact test.

### Immunohistochemistry analysis and chromosomal translocation abnormalities

**Fig 2:**
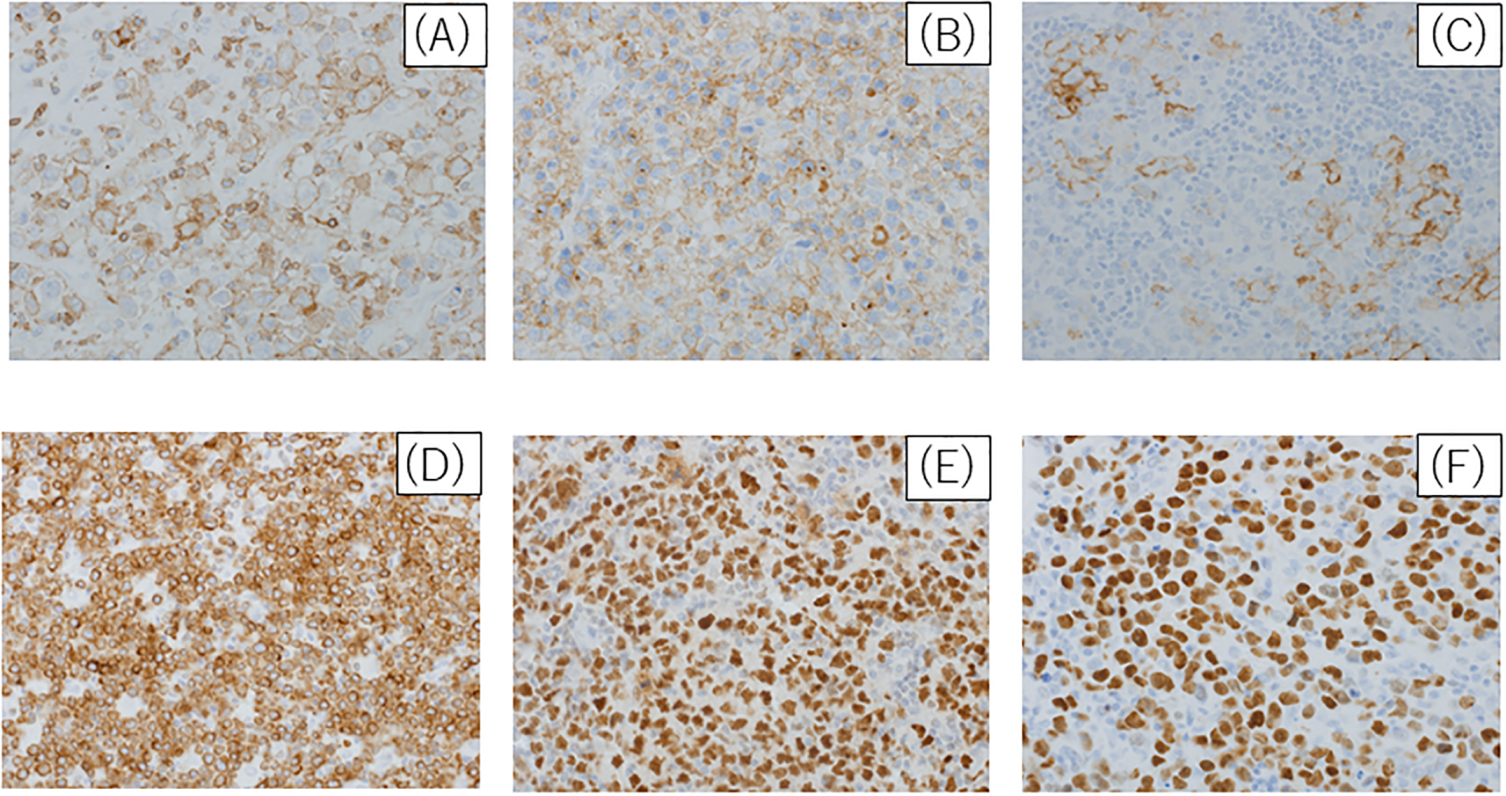
Analysis of immunohistochemistry staining in polyploid DLBCL. Tumor cells were positivity in each immunohistochemistry. CD5 (X400), (B) CD10 (X400), (C) CD30 (X400), (D) BCL2 (X400), (E) BCL6 (X400) and (F) MUM1 (X400).

In order to examine the characteristics of immunohistochemistry analysis and chromosomal translocation abnormalities. The IHC characteristics and chromosomal translocation abnormalities between the two groups are summarized in Table 3. Fig 2 shows the result of IHC in the polyploid DLBCL cases. No significant difference was observed between polyploid DLBCL and control DLBCL cases with respect to the cell of origin according to Hans classification markers (CD10, BCL6, and MUM1) [17] and the other markers (CD5, BCL2, CD30, and EBV-ISH). As shown in S1 Fig, there was no significant difference in the proportion of positivity of various markers among CD20-positive tumor cells between the two groups. In addition, there was no significant difference between polyploid DLBCL and control DLBCL cases with respect to the rates of *BCL2*, *BCL6*, and *MYC* translocations, respectively.

**Table 3:** Immunohistochemistry analysis and chromosomal translocation abnormalities.

	Polyploid cases (N = 51)		Control cases (N = 53)		*p*-value
	No.	%	No.	%	
Immunohistochemistry					
CD5 expression	12/51	23.5	14/53	26.4	0.73
CD10 expression	18/51	35.3	17/53	32.1	0.73
CD20 expression	51/51	100	53/53	100	1.00
BCL2 expression	40/51	78.4	38/48	79.2	0.93
BCL6 expression	38/51	74.5	31/53	58.5	0.08
MUM1 expression	36/51	70.6	39/53	73.6	0.73
CD30 expression	6/51	11.8	6/53	11.3	0.94
EBER positivity	2/51	3.9	5/53	9.4	0.44*
Cell of origin					
GCB type	23/51	41.2	19/53	35.8	0.34
Non-GCB type	28/51	58.8	34/53	64.2	
Chromosomal translocation abnormalities					
*BCL2* translocation	8/51	15.7	4/53	7.5	0.23*
*BCL6* translocation	6/51	11.8	10/53	18.9	0.31
*MYC* translocation	2/51	3.9	2/53	3.8	1.00*

GCB, germinal center B-cell-like diffuse large B-cell lymphoma; EBER, EBV-encoded small RNAs

*Fisher’s exact test.

### Clinical follow-up

**Fig 3:**
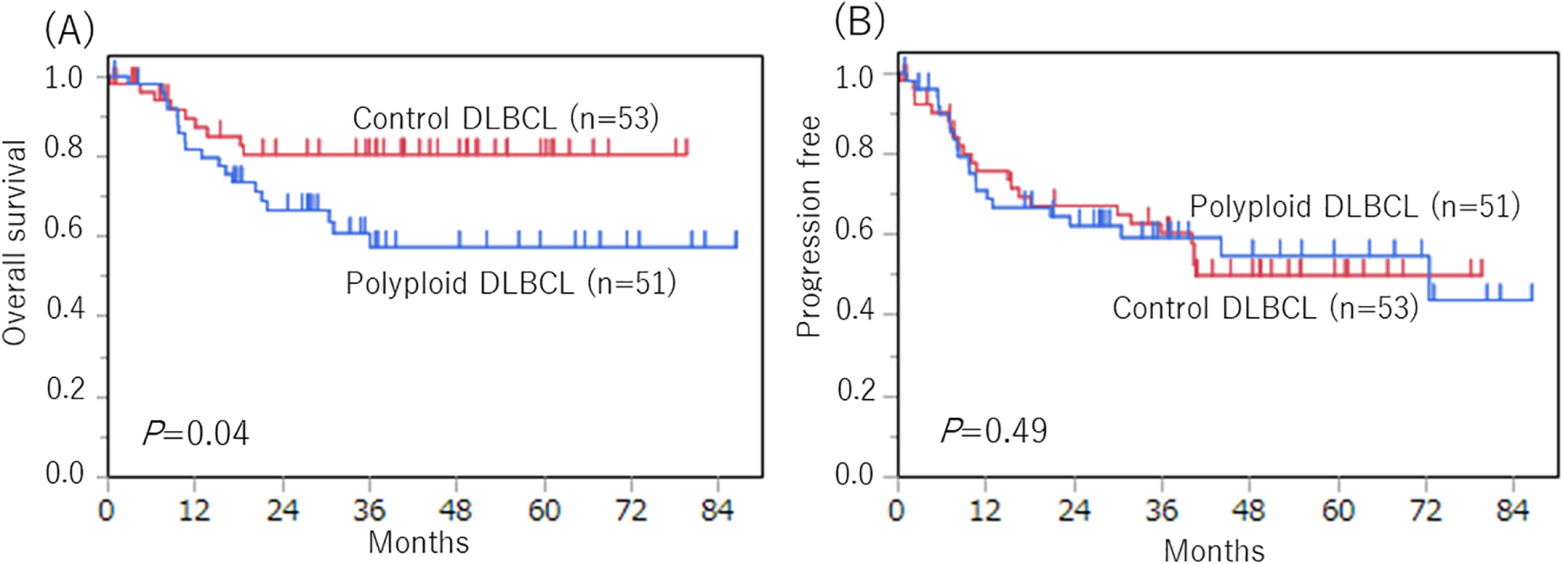
Overall survival and progression-free survival between polyploid DLBCL, NOS (n = 51) and control DLBCL, NOS (n = 53) patients. (A) The overall survival curves were significantly worse for polyploid DLBCL than control DLBCL (*p* = 0.04). (B) The progression-free survival curves were not significantly different between the polyploid DLBCL and control DLBCL (*p* = 0.49).

**Fig 4:**
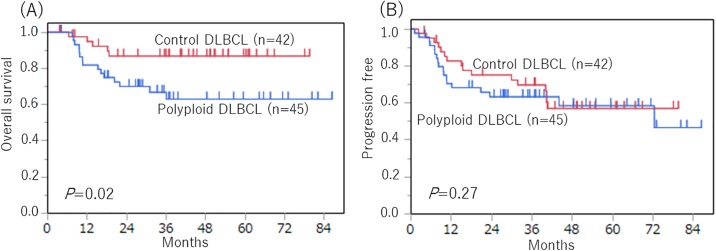
Overall survival and progression-free survival between polyploid DLBCL, NOS (n = 45) and control DLBCL, NOS (n = 42) patients in only cases who received R-CHOP regimens. (A) The overall survival curves were significantly worse for polyploid DLBCL than control DLBCL (*p* = 0.02). (B) The progression-free survival curves were not significantly different between polyploid DLBCL and control DLBCL (*p* = 0.27).

We show the OS and PFS of polyploid DLBCL and control DLBCL cases. (Fig 3) The OS curve was significantly poorer in the polyploid cases than in the control cases (*p* = 0.04). The PFS curves were not significantly different between the polyploid DLBCL and control DLBCL (*p* = 0.49). In addition, we further compared the OS and PFS between the two groups including only cases that received R-CHOP regimens to more effectively investigate the effect of polyploidy on the treatment response. As shown in Fig 4, OS was significantly poorer in the polyploid cases than in the control cases (*p* = 0.02), whereas there was no difference in PFS between the two groups (*p* = 0.27).

### Prognostic factors

We conducted univariate and multivariate analysis to investigate factors related to OS in DLBCL. As shown in Table 4, the univariate analysis revealed the following results: polyploid abnormalities (hazard ratio: HR, 2.19 [95% confidence interval: 95% CI, 0.99–4.86], *p* = 0.05), and IPI high int or high risk (HR, 4.32 [95% CI, 1.47–12.74], *p* = 0.008). The multivariate analysis indicated that polyploid abnormality (HR, 3.11 [95% CI, 1.27–7.66], *p* = 0.01) was an independent poor prognostic factor of OS.

**Table 4:** Univariate and multivariate analysis for overall survival in DLBCL.

	Parameters	Hazard ratio [95% confidence interval]	*p*-value
Univariate analysis			
	Polyploid abnormalities	2.19 [0.99–4.86]	0.05
	B symptoms	1.53 [0.65–3.59]	0.33
	CD5 expression	1.49 [0.67–3.30]	0.32
	BCL2 expression	1.66 [0.68–4.08]	0.27
	IPI high int or high risk	4.32 [1.47–12.74]	0.008
	Non-GCB subtype	1.45 [0.64–3.30]	0.37
Multivariate analysis			
	IPI high int or high risk	5.36 [1.80–15.92]	0.003
	Polyploid abnormalities	3.11 [1.27–7.66]	0.01

IPI, International Prognostic Index; GCB, Germinal center B-cell-like diffuse large B-cell lymphoma.

## Discussion

In our previous study, we found unique pathological features of patients with polyploid DLBCL, presenting a greater number of huge and multinucleated cells compared to those of the control group (DLBCL without polyploidy) [11]. In the current study, we demonstrated that polyploid DLBCL represents a poor prognostic factor, with a reduction in OS and emerging as an independent factor contributing to poor prognosis in multivariate analysis.

Abnormalities of *p53* have been reported to cause polyploid chromosomal abnormalities [18,19]. Tetraploidization promotes tumor formation in the mammary gland epithelial cells of mice with non-functional *p53*, and it is believed that *p53* gene abnormalities play an important role in the formation of polyploid chromosomal abnormalities [5]. A previous study has demonstrated that immunostaining of p53 (>50% positive in tumor cells) is useful as an alternative means to predict the mutation of *p53* in DLBCL [20]. In our study, 81.8% (27/33) of polyploid DLBCL cases tested positive for p53 in the immunohistochemistry analysis. Although the cut off (>30% positive in tumor cells) was different among the various studies, 38.4%–48.4% of DLBCL cases [21–23] have been reported to test positive for p53. Compared with these previous reports, more p53-positive polyploid DLBCL cases were found in the current study. However, in our study, mutation analysis of *p53* was not performed, and the number of cases with a confirmed *p53* mutation is unknown. Together, these results indicate that mutation of *p53* may play an important role in the formation of polyploid chromosomal abnormalities in polyploid DLBCL.

In our study, polyploid DLBCL was associated with a significantly worse overall survival only in cases who received R-CHOP regimens. This might be attributed to drug resistance. In a previous study using a colon cancer cell line, tetraploid cells were found to be associated with drug resistance caused by chromosomal instability [24]. It was reported that there is an association between chromosomal instability and intratumor genetic heterogeneity in gastric cancer and colorectal cancer with aneuploidy, including polyploidy [25,26]. Intratumor genetic heterogeneity may play a role in drug resistance [27,28]. By using array CGH, intratumor genetic heterogeneity was detected in DLBCL, adult T-cell leukemia/lymphoma, mantle cell lymphoma, and peripheral T-cell lymphoma, and all of these malignant lymphomas with intratumor genetic heterogeneity were resistant to treatment [29,30]. Based on these results, we hypothesize that poorer OS in polyploid DLBCL is caused by drug resistance thorough chromosomal instability and intratumor genetic heterogeneity; however, more detailed research is necessary to validate our speculation.

In the multivariate analysis, polyploid chromosomal abnormality was found to be an independent poor prognostic factor. Generally, solid tumors (including esophagus carcinoma, and ovarian carcinoma) with polyploid chromosomal abnormalities have a poor prognosis [31,32]. Chromosomal instability resulting from ongoing numerical and structural chromosomal aberrations might be responsible for this effect [31,32]. Further, there are reports that the complex karyotypes that can be detected with G-banding are related to chromosomal instability [33,34]. Chromosomal instability in solid tumors is strongly correlated with progression to high-grade transformation and is generally considered a poor prognostic factor [35]. In a study of colon cancer, long-term culture and examination of stable diploid progenitors by using isogenic tetraploid cells showed that chromosomal instability was induced after polyploidization (genome-doubling), and polyploid abnormalities were caused at a comparatively early stage in the examination [36]. These findings suggest a close relationship between polyploid chromosomal abnormalities and chromosomal instability, indicating that polyploidy may cause poor prognosis in DLBCL cases.

This study has some limitations. The cases in the current study were classified into polyploid cases and cases without polyploidy by using G-banding. The karyotypes were not necessarily obtained for all cases with G-banding for various reasons, including the status of the lymphoma cells and quality of tissue samples. Therefore, the possibility of some bias in case selection cannot be excluded. Nonetheless, the G-banding technique is highly versatile and is widely used in the diagnosis of hematopoietic tumors. As such, the detection of polyploid abnormalities in a G-band examination is a useful tool in prognosis. Detailed genetic examination in the future should be performed to ascertain the results in this study.

In conclusion, polyploid chromosomal abnormality was associated with poor OS and was an independent poor prognostic factor. The detection of polyploid DLBCL by using G-banding might represent a new prognostic factor of poor OS.

## Supporting information

S1 Fig Quantification of various markers in CD20-positive tumor cells. CD5, (B)CD10, (C)CD30, (D)BCL2, (E)BCL6, (F)MUM1. There was no significant difference in the proportion of positivity of various markers in CD20-positive tumor cells between the two groups.
(TIF)

S1 File Clinical data in this study. (XLSX)

S1 Table Karyotype of polyploid DLBCL. (DOCX)

S2 Table Karyotype of control DLBCL. (DOCX)

S3 Table Antibodies used for flow cytometric immunophenotyping analysis and immunohistochemical staining analysis. (DOCX)
