# Supplementary figures and images for: Clinicopathological analysis of polyploid diffuse large B-cell lymphoma
Source: PLoS One. 2018 Apr 11;13(4):e0194525. doi: 10.1371/journal.pone.0194525 (PMC5894967; doi:10.1371/journal.pone.0194525)

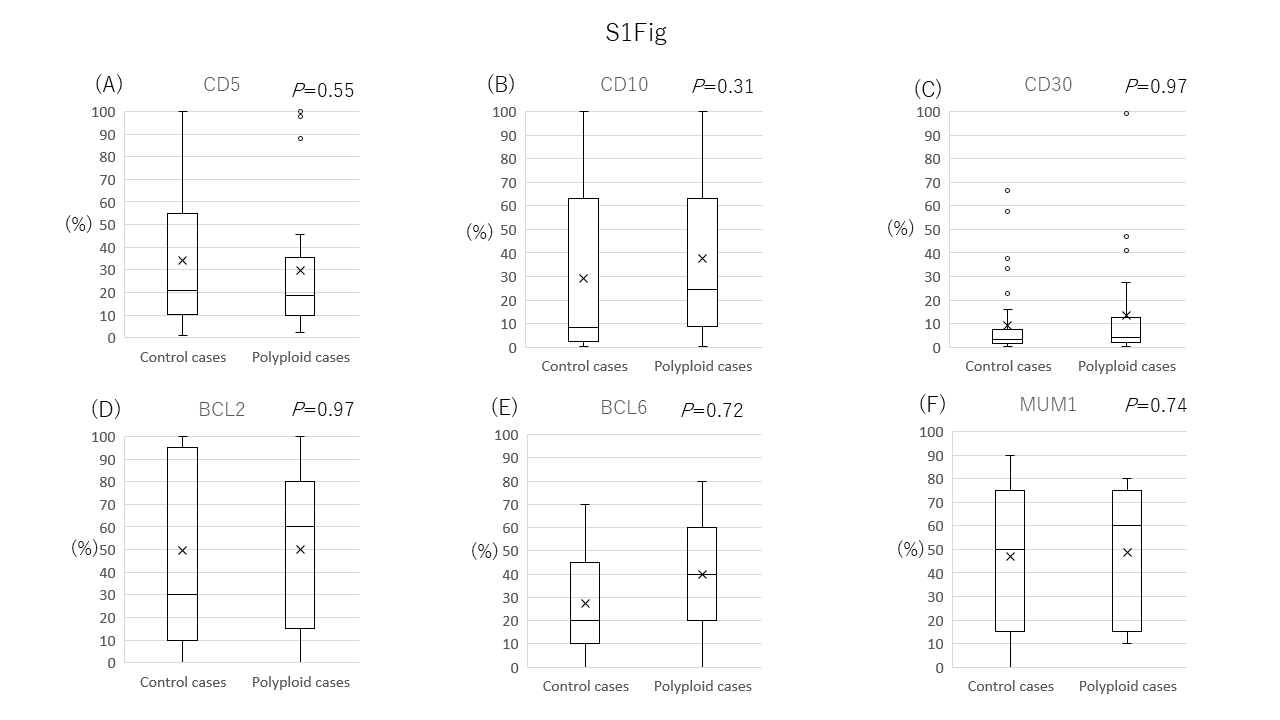

Supplement: S1 Fig — CD5, (B)CD10, (C)CD30, (D)BCL2, (E)BCL6, (F)MUM1. There was no significant difference in the proportion of positivity of various markers in CD20-positive tumor cells between the two groups. (TIF) [file pone.0194525.s001.TIF]
