# Supplementary material for: Clinicopathological analysis of polyploid diffuse large B-cell lymphoma
Source: PLoS One. 2018 Apr 11;13(4):e0194525. doi: 10.1371/journal.pone.0194525 (PMC5894967; doi:10.1371/journal.pone.0194525)
Supplement: S1 Table — (DOCX) [file pone.0194525.s003.docx]

Supplementary Table 1. Karyotype of polyploid DLBCL.

| Case | Karyotype |
| --- | --- |
| 1 | 90,XXX,-X,add(1)(p11),+5,-7,-8,-8,dup(12)(q13q24.1)X2,-14,-15,-15,-17,add(18)(q21),-20,+6mar[3] 46,XX[17] |
| 2 | 85-89,XXXX,-1,add(1)(q21),-2,-3,-4,-4,-7,-9,-10,+12,-13,-14,-15,-16,-20,+9mar[cp7] 46,XX[4] |
| 3 | 89-93,XX,-Y,-Y,add(1)(p11),add(1)(p11),add(2)(q31)X2,-4,del(6)(q?)X2,add(7)(q22)X2,-10,+13,  -14,+16,-17,-17,+19,-21,+6mar[cp4] |
| 4 | 46,X,?insinv(Y)(p11.2q11.2),t(2;3)(q37;q21)[1/20] 47,X,?ins(Y),+?inv(Y)[1]  92-94,XX,+X,-Y,?inv(Y),ins(1;?)(q21;?)X2,-2,-6,-7,add(9)(q34),-11,+12,-14,-14,-15,+19,-21,-22,+7mar[cp]  46,X,?inv(Y)(p11.2q11.2)[12] |
| 5 | 84-90,XX,-Y,-Y,-1,add(3)(q21)X2,+5,-6,-8,-9,-10,add(10)(q22),-11,+12,-14,t(14;18)(q32;q21),  -17,+der(18)t(14;18),-22,-22,+7mar[cp5] |
| 6 | 49,XX,+1,der(1;13)(q10;q10),-2,+add(3)(p11),t(3;14;18)(q27;q32;q21),der(3)t(3;14)(q27;q32),+7,  der(8;9)(q10;q10),add(9)(p11),add(12)(p11.2),add(14)(q22),-22,+mar1,+mar2,+mar3,+mar4[1]  82-83,XXX,-X,+1,der(1;13)(q10;q10)X2,-2,-2,t(3;14;18)(q27;q32;q21),der(3)t(3;14)(q27;q32)X3,  -4,+7,+7,der(8;9)(q10;q10)X2,add(9)(p11)X2,-11,+add(12)(p11.2),add(12)(q13)X2,-13,-14,add(14)(q22)X2,  -15,-15,-16,-16,der(18)t(14;18)(q32;q21),-19,-20,-21,-22,-22,-22,+mar1,+mar2X2,+mar3X2,+3ma[cp2] 46,XX[17] |
| 7 | 90-92,XXY,-Y,-2,-3,-4,-5,-6,+add(9)(p22),-13,-14,-14,-15,-15,-18,add(18)(q21),+11mar[cp2] 46,XY[2] |
| 8 | 85-100,XXYY,del(1)(p?),-3,+5,-6,-6,+7,-9,-9,+10,add(12)(q24.1)X2,-15,+20,+7mar[cp3] 46,XY[13] |
| 9 | 90-95,XXXX,-1,+3,+3,+6,add(6)(q11)X2,del(7)(q?)X2,+del(7)X3,-8,-12,-14,-15,-17,add(17)(p11.2),  -19,add(20)(q13.1),del(20)(q1?),-22,+marlX2,+mar2[cp20] |
| 10 | 78-88,XXYY,add(1)(p21),-2,-4,-6,add(8)(p11.2)X2,-9,-11,-11,-13,-13,-13,der(15;19)(q10;q10)X2,-17,-18,  -21,-22,+11mar[cp19], 46,XY[1] |
| 11 | 89-92,XX,-X,-X,t(3;22)(q27;q11.2)X2,-4,-4,del(6)(q?)X2,add(7)(q32)X2,+18,+18,i(18)(q10)X4,  -19,+mar[cp8] 46,XX[2] |
| 12 | 78-88<3n>,XX,+X,-Y,-1,add(1)(p11),-2,add(2)(q11.2),-3,add(3)(q27),+add(4)(q21),+del(6)(q?),i(6)(p10)X2,  +add(7)(q11.2),-8,add(11)(q23),add(11)(q23),-14,-14,-15,-17,add(18)(q21),add(18)(q21),+17mar[cp7] 46,XY[7] |
| 13 | 84<3n>,XX,-X,add(1)(p34),+del1(p?)X2,-2,add(3)(q27),-4,-5,del(6)(q?),+8,add(8)(q24)X2,+11,+12,  -14,t(14;18)(q32;q21),-15,+der(18)t(14;18),+19,+20,+21,+12mar[2] 46,XX[18] |
| 14 | 78-88<3n>,-Y,add(X)(q22),der(X)add(X)(p11.2)add(X),+der(1)add(1)(p32)dup(1)(q32q12),  dup(1),+add(3)(p13),+add(5)(p11),+del(5)(q?),del6(q?),+7,+7,+add(7)(q22),-8,-8,+9,  -10,+11,dup(11)(q13q23)X2,+12,+12,+14,-15,-15,-15,+18,-19,+14mar[cp15] 46,XY[5] |
| 15 | 46,X,-X,add(2)(p21),del(2)(q?),+mar1[13]  89,slX2,add(3)(q11.2)X2,-7,-8,-8,-9,-13,-mar1,+3mar[4] 46,XX[3] |
| 16 | 103-110<4n>,XXX,+X,+X,+X,-1,-4,-5,del(6)(q?)X2,+11,+15,-17,-17,+22,+11,mar[cp6] 46,XX[14] |
| 17 | 87-92,XXX,+X,-Y,+add(1)(p11),+add(1)(q11),+2,-6,-6,-8,der(9)t(9;14)(p11;q11.2),-10,-12,-13,-13,-14,  -14,-15,+16,-17,-21,-22,+7mar[cp7] 46,XY[1] |
| 18 | 76-85,XX,-Y,+add(1)(p13),add(2)(q11),+3,+3,add(3)(p13)X3,add(4)(q11),-5,add(5)(q11)X2,-6,-7,+8,  -10,+11,+11,+12,+14,t(14;18)(q32;q21)X2,-15,add(17)(p11),+18,+19,add(19)(q11)X2,+20,+5mar[cp13] 46,XY[7] |
| 19 | 80-84<3n>,YY,+addd(X)(q11),add(X)(q11),+add(2)(p11.2),add(3)(q27)X2,+der(3)(del(3)(p?)add(3),+5,  -6,-6,+7,+8,-10,-11,-12,-13,+16,+17,add(19)(q13.1),+20,+20,+21,+21,+21,add(22)(q13),+6mar[cp4] 46,XY[16] |
| 20 | 84-85,XXY,-Y,add(1)(p11),add(1)(p11),-5,add(5)(p11),-6,-8,-9,-10,add(11)(p15),-12,-13,-13,  -14,t(14;18)(q32;q21),-15,-17,-17,-18,add(18)(q21),-22,+7mar[cp2] 46,XY[6] |
| 21 | 99-100,XX,-X,-X,del(2)(q?)X2,+5,del(6)(q?),+7,+7,-8,-9,+10,+11,-18,-18,-19,-19,-19,+20,-22,+12mar[cp3] 46,XX[17] |
| 22 | 84-86<2n>,XX,+3,add(3)(q21)X2,+5,+6,del(6)(q?)X2,-8,del(11)(p?),+19,+21,+36mar[cp2] 46,XX[1] |
| 23 | 80-90,XX,+X,+X,-Y,-Y,-1,-2,-3,add(3)(p11),-5,-6,-6,del(8)(q24)X2,-9,-10,t(14;18)(q32;q21)X2,-17,-17,-17,  -19,-21,-21,-22,-22,+der(?)t(?;1)(?;q21),+5mar[cp6] 46,XY[1] |
| 24 | 82-86,X,-X,-Y,-Y,;i(1)(q10),add(2)(p13),-4,-4,add(4)(q31),-6,add(6)(q13),+7,-9,-10,-13,add(13)(p11.2),-14,  -15,-15,-18,-19,+20,+mar1,+mar2,+mar3[cp13] 85,idem,+mar4[2] 46,XY[5] |
| 25 | 89-91,XX,+X,-Y,-Y,i(1)(q10),-2,-2,add(2)(p13),der(3)add(3)(p21)t(3;14)(q27;q32),t(3;14),  -4,idic(6)(p21),idic(6)(q11)X2,-8,+12,add(12)(q22)X3,+der(14)t(3;14),-15,add(15)(p11.2),-18,  -20,+r,+3mar[cp7] 46,XY[2] |
| 26 | 79-98,XXXX,+X,add(1)(p11)X2,add(1)(p11),-3,add(3)(q21)X2,-6,-6,-6,-8,-8,add(9)(p11),+11,-13,-13,-13,-14,  -14,add(14)(q32),+15,-16,-17,+18,-19,-19,add(19)(q13.1),add(19)(q13.1),+20,-21,-22,+6mar[cp10] |
| 27 | 88-93,XXY,-Y,add19(q21)X2,+2,-3,-4,add(4)(q21),-5,del(6)(q15)X2,-8,-8,-9,add(9)(q22),-10,  -12,add(12)(p11.2),add(14)(q32)X2,-15,-17,+18,+21,+7mar[cp12] 46,XY[2] |
| 28 | 46,XY,add(1)(q21)[1]  91-94,XXYY,-1,der(1;21)(p10;q10),+3,add(3)(q27)X2,-4,-5,-6,-7,-11,-12,-17,+18,+18,  der(19)?t(1;19)(q23;p13.3)X2,-22,+7mar[cp4], 46,XY[7] |
| 29 | 99-100,XXXX,+X,+add(1)(p11),add(1)(p11),add(1)(q21),-2,-2,-3,-4,-5,-8,-8,+9,-13,-13,t(14;18)(q32;q21)X2,  -16,-17,+18,-21,-22,-22,+17mar[cp6] 46,XX[9] |
| 30 | 89-90,XXY,-Y,add(2)(q11.2)X2,add(5)(p13),-6,-6,-6,-8,-8,-11,add(14)(q32),t(14;18)(q32;q21)X2,-15,-16,  -17,+7mar[cp2] |
| 31 | 83-91,XXX,-X,-1,-4,-4,+5,+6,der(6;18)(p10;q10)X3,-8,-9,-10,-11,add(11)(q23)X2,  -12,add(12)(q11),add(14)(q22)X2,+16,+16,-17,add(17)(p11.2),+18,+20,+der(?)t(?;1)(?;q21)[cp6] 46,XX[14] |
| 32 | 93-94,XXYY,+X,i(1)(q10),-2,t(2;8)(p12;q24),-3,-4,-6,del(6)(q?)X2,+7,-8,der(8)t(2;8),-9,+12,-15,  -18,+6mar[cp3] |
| 33 | 72-85<3n>,XYY,der(19(del(1)(p?)add(1)(q32),add(2)(p13),add(3)(q11.2),del(4)(q?),+6,del(6)(q?)X2,  del(7)(p13)X2,+12,add(12)(p11.2)X2,+14,+17,+add(18)(q21),+19,+20,del(20)(q1?)X2,+21,+8mar[cp13] 47,XY,+Y[1] 46,XY[3] |
| 34 | 93-101,XXYY,+X,add(1)(q32)X2,+3,+3,+3,+5,add(6)(p21)X2,+7,-8,add(8)(p11.2),add(8)(p11.2),  add(8)(p11.2),-14,-14,-15,-15,-17,-17,-18,-18,-22,+13,mar[cp16] 46,XY[4] |
| 35 | 92-100,XX,-X,-X,add(1)(q32)X2,del(2)(q?)X2,t(3;8)(q13;p22)X2,+5,del(6)(q?)X2,+7,+7,ins(7;?)(q22;?)X4,+8,  der(8)t(3;8)X2,-10,add(11)(q23)X2,+18,+18,-20,-21,+marlX2[cp19] 97.idem,-5,+8,+mar2X2[1] |
| 36 | 86-96,XXYY,+X,+X,+X,+X,-1,add(1)(q21),t(1;18)(q32;q21)X2,-2,+add(3)(q11.2),add(4)(q21)X2,  del(6)(q13)X2,-7,add(7)(q32),+8,-9,?t(9;14)(p13;q32),add(10)(p11.2),add(10)(q22),-11,add(11)(q13),+12,-13,  -13,-14,-14,der(14)?t(9;14),+15,del(17)(p?)X2,-19,-19,-21,+8mar[cp19] 46,XY[1] |
| 37 | 97-101,XXXX,+X,+2,t(3;14)(q27;q32)X2,+5,+6,del(6)(q11)X2,+7,+12,+12[cp20] |
| 38 | 77-91,XXYY,+X,+add(1)(p11),i(1)(q10),der(3;6)(q10;p10),-4,-7,-8,t(8;22)(q24;q11.2),-10,-12,-14,-14,-16,-18,  -19,+7mar[cp18] 46,XY[2] |
| 39 | 81-92,XXXX,+X,-1,-1,add(1)(q21),-2,+add(3)(q27),-4,-5,add(5)(q31)X2,der(6)t(1;6)(q21;q11)X2,-9,-10,  -11,add(12)(q24.1),add(14)(q32)X2,-16,-17,-18,-18,add(19)(p13),+der(?)t(?;1)(?;p13),+8mar[cp6] 46,XX[3] |
| 40 | 87-92,XXXX,-2,del(6)(q?)X2,+7,-8,-8,-9,-12,add(14)(q24),-15,add(17)(p11.2)X2,-19,-21,  -22,+mar1,+mar2,+mar3[cp20] |
| 41 | 89-91,XXYY,-1,-1,-1,add(2)(p12)X2,add(3)(q27)X2,+5,add(6)(q13)X2,-7,-8,-9,ad(9)(p13)X2,add(10)(p11.2),  -12,-12,-13,-13,der(14)t(1;14)(q21;q32)X2,-15,-15,-16,-17,-17,-18,-18,-22,+14mar[cp3] 46,XY[6] |
| 42 | 93-97,XY,add(1)(p11),dup(1)(q21q32),+3,add(9)(p11),der(9)add(9)(p11)del(9)(q?),add(10)(p11.2),  -14,add(14)(q32),add(17)(p11.2),+mar1[cp14] 96,idemX2,+X,+1,-add(1)X2,+9,+9,-add(9)X2,+add(9)(p11),  -der(9)add(9)del(9),+10,+10,-add(10)X2,+13,+mar[1] 46,XY[2] |
| 43 | 91-95,XX,-Y,-Y,-1,-2,add(3)(p13)X2,-4,-4,-10,del(11)(q?)X2,add(13)(q32)X2,t(14;18)(q32;q21)X2,  add(15)(q22)X2,-16,-17,-17,-17,-17,+3mar[cp14] 46,XY[3] |
| 44 | 93-106,XXYY,+X,-1,-1,-2,ad(2)(q11.2),-3,-3,add(3)(p21),-5,del(6)(q?),-7,add(7)(q32)X2,-9,-9,-10,+11,-13,-14,  -14,-15,-15,-15,+16,+16,add(16)(q22)X3,-17,-17,-17,add(18)(q21)X2,-21,-22,  -22,+der(?)t(?;14)(?;q11.2),+21mar[cp7] 46,XY[7] |
| 45 | 90-92,XXXX,-1,-6,-8,-10,-12,t(14;18)(q32;q21),-19,-22,+5mar[cp6] 46,XX[8] |
| 46 | 75-86<3n>,XXX,-2,+add(3)(q11.2),+add(3)(q21),-4,-4,+5,-6,add(6)(q13),+7,add(7)(q22)X2,-8,-9,+10,  add(11)(q13)X2,-12,-13,-13,+14,-15,-16,-17,+18,+18,+19,+20,add(20)(q11.2)X2,+21,+22,+16mar[cp19] 46,XX[1] |
| 47 | 79-81<3n>,XX,-Y,add(1)(q32),del(2)(q?),-4,+5,del(6)(q?),-7,add(7)(q22),del(8)(q?)X2,+9,-10,+add(11)(p11.2),  -13,+14,+15,+16,add(17)(p11.2),-18,add(19)(p13),+21,-22,-22,+13mar[cp7] 46,XY[4] |
| 48 | 92-94,XXXX,der(1;12)(p10;q10)X2,+2,-3,add(3)(q11.2),+7,-9,add(11)(p11.2),-13,+16,-17,  -18,+20,+20,+21,+2mar[cp4] |
| 49 | 88-95,XX,der(Y)t(Y;1)(q12;q21)X2,-1,t(1;16)(p13;q13)X2,-2,add(3)(q21)X2,add(4)(q11),del(6)(q?)X2,-7,  -8,add(19)(p13)X3,+3mar[cp15] 46,XY[5] |
| 50 | 89,XXXX,-1,del(2)(q?)X2,add(3)(q21)X2,-4,-6,-8,-8,add(11)(q23)X2,add(15)(p11.2),add(16)(q12.1)X2,  add(17)(p11.2)X2,add(22)(p11.2)X2,+mar1X2[15] 46,XX[5] |
| 51 | 72-87,XX,-Y,add(1)(q21),-5,+6,del(6)(q13)X2,add(7)(q22),-8,-9,-9,-10,+12,add(13)(p11.2),+15,+16,  -17,add(19)(q13.1),+20,-21,-21,-21,+16mar[cp15] 46,XY[1] |
