# Supplementary material for: Clinicopathological analysis of polyploid diffuse large B-cell lymphoma
Source: PLoS One. 2018 Apr 11;13(4):e0194525. doi: 10.1371/journal.pone.0194525 (PMC5894967; doi:10.1371/journal.pone.0194525)
Supplement: S2 Table — (DOCX) [file pone.0194525.s004.docx]

Supplementary Table 2. Karyotype of control DLBCL.

| Case | Karyotype |
| --- | --- |
| 1 | 45,XX,add(1)(p13),add(3)(q21),-6,-7,+mar[2] 46,XX[4] |
| 2 | 44-46,X,-X,add(1)(q21),-2,add(3)(p25),+del(6)(q?),-8,add(9)(p11),add(10)(q22),+11,-17,+18,+19,-20,add(21)(p11.2),-22,+r,+mar[cp7] 46,XX[13] |
| 3 | 58<2n>,X,+X,-Y,del(5)(q?),+6,+10,+11,+12,+16,-17,-18,+19,add(19)(q13.1)X2,+20,-22,+8mar[2] 46,XY[5] |
| 4 | 47-48,X,+X,+12,+13[cp9] 47,sl,-13,+21[6] 48,sl,+21[5] |
| 5 | 46,XX,t(1;7)(p22;p11.2)[2] 48,XX.+5,+7[2] 47,XX,+X[1] 47,XX,+18[1] 46,XX[14] |
| 6 | 48,XY,+X,dup(1)(q21q32),+3,del6(q?)[9] |
| 7 | 49-51,XY,del(1)(p?),add(2)(q31),+add(3)(p21),+5,der(10;17)(q10;q10),+13,add(16)(p11.2),+18[cp8] 50,sl,add(14)(q32),del(15)(q?),+16,-add(16),+mar1[8] 46,XY[2] |
| 8 | 44-50,X,-Y,-1,+3,+7,-9,-9,-15,+mar1,+mar2[cp6] 45,sl,add(4)(p16)[2]  45,sl,t(2;15)(q35;q15)[2] 46,XY[10] |
| 9 | 48,X,-X,add(1)(p32),add(1)(q32),+3,del(3)(p13p21)X2,+6,add(7)(q22),add(7)(q32),add(12)(q13),  add(14)(q22),add(19)(q13.1),+21,del(22)(q?)[10] 49,sl,+20[1] 46,XX[9] |
| 10 | 68-71,XX,-X,+1,-2,add(4)(p16)X2,-6,-8,+9,add(9)(p13)X2,-13,+add(14)(q32),-16,+18,-20,+5mar[cp4] 46,XX[1] |
| 11 | 47,-X,add(X)(p11.2),add(1)(p32),add(6)(q13),+der(12)add(12)(p11.2)add(12)(q24.1),add(13)(q32),  add(17)(p11.2),-22,+mar1,+mar2[12] 47,sl,-add(6),+add(6)(q13)[3] 49,sdl1,+7,+10[2] 46,XX[3] |
| 12 | 45,X,-Y,add(1)(p11),add(2)(p13),t(3;14)(q27;q32),del(5)(p?)[4] |
| 13 | 50,XX,+3,der(6)add(6)(p21)del(6)(q?),+add(7)(q22),+18,+add(19)(p13),add(19)(q13.1)[19] 46,XX[1] |
| 14 | 49-57,-Y,der(X)t(X;11)(p22.3;q13),add(19)(q21),+3,der(13)t(11;13)(q13;p11.2),add(17)(p11.2),  +18,+mar1,+mar2,+mar3[cp17] 52,sl,+mar2,+mar3[3] |
| 15 | 47,XX,inv(3)(p25q29),del(6)(q?),+add(7)(p15),add(9)(p13),t(14;18)(q32;q21)[20] |
| 16 | 47,XX,+4[3] |
| 17 | 46,X,-Y,der(2)add(2)(p13)add(2)(q33),+7,i(8)(q10),+19,-21,add(22)(p11.2)[18] 47,sl,+19[2] |
| 18 | 45-47,X,-Y,-1,der(1)add(1)(p34)add(1)(q21),add(3)(q11.2)X2,der(4)t(1;4)(p13;q21),add(7)(q11.2),  add(8)(q22),-12,-14,+18,-19,add(20)(q11.2),+der(?)t(?;14)(?;q11.2),+r1,+mar1[cp4] 46,sl,+9[16] |
| 19 | 53-54,XY,+X,-2,-2,-3,add(4)(p11),del(6)(q?),-9,add(9)(p13),add(12)(p11.2),-16,+18,-19,-20,+13mar[cp5] 46,XY[15] |
| 20 | 47,XX,+12[8] 46,XX[1] |
| 21 | 49,XX,add(1)(p11),add(4)(p16),+6,+7,add(13)(p11.2),add(19)(q13.1),+21[1]  49,sl,del(19)(p13)[7] 46,XX[1] |
| 22 | 46,XX,add(6)(p23),add(12)(q13),add(14)(q32)[14] 46,sl,add(15)(p11.2)[3] 47,XX,+9[1] 46,XX[2] |
| 23 | 46,XY,t(3;13)(q27;q14),del(6)(q?),t(8;14)(q24;q32),ins(12;?)(q13;?)[8] 46,XY[12] |
| 24 | 48,X,add(X)(q13),add(1)(p11),+add(3)(q27),add(6)(p11),-15,+18,-21,+mar1,+mar2[4] |
| 25 | 51,XY,add(3)(q27),add(4)(q21),+del(6)(q?),del(6)(q?),-9,add(9)(p11),-13,  -14,add(18)(p11.2),+mar1,+mar2,+mar3,+mar4,+mar5,+mar6,+mar7[7] |
| 26 | 48,XX,t(3;14)(q27;q32),+5,add(6)(q13),+7,del(15)(q?),del(19)(p13)[12]  49,idem,+mar1[3] 50,idem,+13,+16[1] 46,XX[2] |
| 27 | 53-54,XX,add(1)(p36.1),add(3)(q21),t(8;14)(q24;q32),+9,der(9;14)(q10;q10),+add(10)(q22),+11,+12,+16,  -17,+20,+21,+mar1,+mar2[cp5] 53,idem,del(4)(q?)[9] |
| 28 | 53,XY,t(1;3)(p22;q27),+i(6)(p10),-8,+9,+12,del(20)(q1?),+mar1,+mar2X2,+mar3X2[5] |
| 29 | 58-60<2n>,XX,add(1)(q32),+der(1)(p11)add(1)(q42)X2,+add(3)(p21),+5,+i(6)(p10),add(8)(q24),  +11,+12,+12,t(14;18)(q32;q21),+17,der(18)t(14;18),+der(18)t(14;18),+del(20)(q11.2q13.3)X2,+mar1[cp13] 59,idem,del(5)(q?)[7] |
| 30 | 47,XX,add(1)(p22),+7,t(14;18)(q32;q21),del(15)(q?),add(17)(q25),add(19)(q13.1),  der(21)t(1;21)(p22;q22)ins(21;?)(q22;?)[20] |
| 31 | 47,XY,+X[2] 49,idem,+8,+9[1] 46,XY[9] |
| 32 | 48,XY,+Y,t(3;14)(q27;q32),i(6)(p10),add(13)(q32),+mar1[10] 46,XY[10] |
| 33 | 47,XX,+15[9] 45,X,-X[4] 46,XX[7] |
| 34 | 45,X,-X,add(1)(q11),der(3)add(3)(p21)add(3)(q27),add(6)(p21),i(6)(p10),add(8)(p11.2),-9,  -14,add(18)(q21),+der(?)t(?;1)(?;q21),+mar1[4] 46,XX[4] |
| 35 | 46,XY,add(3)(q27),del(6)(q?),add(9)(p13)[19] 47,idem,+12[1] |
| 36 | 45,XX,-4,del(7)(q22),-8,add(16)(q22),+18[2] 46,sl,+mar1[11] 46,XX[7] |
| 37 | 49-52,XX,+X,+del(1)(p?),der(1;13)(q10;q10),del(3)(p13p21),+5,del(6)(q?),add(7)(q11.2),+add(8)(q22),  +10,add(15)(q22),add(17)(p11.2),add(22)(q11.2)[cp5]  50,sl,add(X)(p22.1)[4/20] 51,sl,+mar1[2] 47,XX,+X[3] 46,XX[6] |
| 38 | 46,X,-Y,add(1)(p11),del(6)(q?),-8,-15,+18,-22,+r1,+mar1,+mar2[4] 47,sl,+18[1] 46,XY[2] |
| 39 | 46,XY,del(3)(q?),der(14)t(3;14)(q27;q32)[20] |
| 40 | 50,XX,+X,+add(2)(q11.2),+7,inv(9)(p12q13),+12[18] 46,XX,inv(9)(p12q13)[2] |
| 41 | 53-55,XX,t(3;22)(q27;q11.2),+12,+13,add(14)(q22),+19,-22,+mar1,+mar2,+mar3,+mar4X2[cp10] 54,sl,+21[3] 55,sdl1,+mar4[3] 46,XX[2] |
| 42 | 47,XX,+X,add(2)(q11.2),del(6)(q?),add(16)(p13.1)[4] |
| 43 | 47,X,-Y,+3,del(5)(q?),add(6)(q15),del(9)(p?),add(17)(q21),+18[4] 46,XY[10] |
| 44 | 43-47,Y,add(X)(p11.2),-1,add(6)(q21),add(7)(p15),add(8)(p11.2),-9,add(12)(p11.2),add(14)(q32),-15,  -16,add(17)(p11.2),+18,+mar1,+mar2,+mar3[cp6] 46,idem,-Y,+add(3)(p21),+12,-add(12)[3]  45,idem,-Y,add(3),-add(8),+12,-add(12),+mar4[3/20] 46,XY[4] |
| 45 | 46,XX,der(3)del(3)(p13q21)t(3;14)(q27;q32),der(14)t(3;14),ins(17;?)(q21;?),add(18)(q21),  der(18)t(1;18)(q12;p11.2)[7] 47,idem,+mar1[11] 46,XX[2] |
| 46 | 48-50,XY,-3,del(4)(q?),-5,del(6)(q?),-8,-9,-13,t(14;18)(q32;q21),-16,-17,-22,+12mar[cp8] |
| 47 | 51,X,+X,-Y,+add(2)(p23),+5,+7,del(8)(q?),+12,+16,add(16)(p13.1)X2,add(21)(p11.2)[14] 46,XY[1] |
| 48 | 46,XX,del(16)(q?)[4] 45,sl,-X[12] 46,sdl1,+18[3] 47,XX,+X[1] |
| 49 | 47,XX,add(1)(p13),add(2)(p13),t(3;14)(q27;q32),+12,add(13)(q14)[10] 47,sl,-12,+21[1] 46,XX[2] |
| 50 | 49-53,XY,+der(3)t(3;12)(p21;q13),+del(9)(q?),add(12)(q24.1),-15,+18,add(19)(q13.1),+mar1[cp4] 53,sl,+X,+3,+4,+11[1] 46,XY[2] |
| 51 | 47,XX,dup(1)(q21q32),add(3)(q21),t(3;14)(q27;q32),-17,+mar1,+mar2[20] |
| 52 | 46-57,XY,-7,-10,-15,+3mar[cp2] 57,XY,-1,-1,+2,+6,+7,+8,-10,+12,-13,-15,-19,+21,+11mar[1] 46,XY[6] |
| 53 | 46-47,X,-X,dup(1)(q32q21),+3,add(6)(q11),add(8)(p11.2),+14,-19,+rl[cp15] 48,idem,+20[1] 46,XX[4] |
