# Supplementary material for: Clinicopathological analysis of polyploid diffuse large B-cell lymphoma
Source: PLoS One. 2018 Apr 11;13(4):e0194525. doi: 10.1371/journal.pone.0194525 (PMC5894967; doi:10.1371/journal.pone.0194525)
Supplement: S3 Table — (DOCX) [file pone.0194525.s005.docx]

Supplementary Table3. Antibodies used for flow cytometric immunophenotyping analysis and immunohistochemical staining analysis.

| Antibodies | Clone | Source | Antigen retrieval | Dilution |
| --- | --- | --- | --- | --- |
| CD5-PE | BL1a | Beckman Coulter | - | 1:100 |
| CD10-PE | ALB1 | Beckman Coulter | - | 1:100 |
| CD20-FITC | B-Ly1 | Beckman Coulter | - | 1:40 |
| CD30-FITC | Ber-H2 | Dakocytomation | - | 1:5 |
| CD45-PerCP | J.33 | Beckman Coulter | - | 1:5 |
|  |  |  |  |  |
| CD5 | 4C7 | Leica Microsystems | EDTA buffer 95℃ 20min | 1:50 |
| CD10 | 56C6 | Leica Microsystems | EDTA buffer 95℃ 20min | 1:100 |
| CD20 | L26 | Dakocytomation | EDTA buffer 95℃ 40min | 1:5 |
| CD30 | Ber-H2 | Dakocytomation | EDTA buffer 95℃ 20min | 1:100 |
| BCL2 | 124 | Dakocytomation | EDTA buffer 95℃ 20min | 1:100 |
| BCL6 | P1F6 | Leica Microsystems | TE buffer 95℃ 40min | 1:20 |
| MUM1 | MUM1p | Dakocytomation | EDTA buffer 95℃ 10min | 1:100 |
| P53 | DO-7 | Dakocytomation | EDTA buffer 95℃ 20min | 1:100 |
